# Light-Controlled ZrO_2_ Surface Hydrophilicity

**DOI:** 10.1038/srep34285

**Published:** 2016-10-05

**Authors:** Aida V. Rudakova, Maria V. Maevskaya, Alexei V. Emeline, Detlef W. Bahnemann

**Affiliations:** 1 Laboratory “Photoactive Nanocomposite Materials”, Saint-Petersburg State University, Ulyanovskaya str. 1, Peterhof, Saint-Petersburg, 198504 Russia

## Abstract

In recent years many works are aimed at finding a method of controllable switching between hydrophilicity and hydrophobicity of a surface. The hydrophilic surface state is generally determined by its energy. Change in the surface energy can be realized in several different ways. Here we report the ability to control the surface wettability of zirconium dioxide nano-coatings by changing the composition of actinic light. Such unique photoinduced hydrophilic behavior of ZrO_2_ surface is ascribed to the formation of different active surface states under photoexcitation in intrinsic and extrinsic ZrO_2_ absorption regions. The sequential effect of different actinic lights on the surface hydrophilicity of zirconia is found to be repeatable and reversibly switchable from a highly hydrophilic state to a more hydrophobic state. The observed light-controllable reversible and reproducible switching of hydrophilicity opens new possible ways for the application of ZrO_2_ based materials.

Real time manipulation of hydrophilic/hydrophobic properties of metal oxide nano-coating surfaces caused by different factors is an attractive topic due to its great practical importance^1^,^2^. The wettability can be switched by alteration of the electric potential, by ultrasonic or thermal treatment, by stress-strain or surface modification, by irradiation of the surface, etc^3^,^4^,^5^,^6^,^7^,^8^. The photo-switching between two different hydrophilic states has been widely explored during the last decade and is generally based on the ability of some solid’s surfaces to become superhydrophilic under UV irradiation (known as the phenomenon of surface photoinduced superhydrophilicity^6^,^9^) and then returning to the initial state in the dark at ambient conditions or after treatment either at slightly elevated temperatures (150 °C–200 °C ) or under IR irradiation^7^,^10^. The vast majority of inorganic semiconductor surfaces, which were studied concerning this matter, are based on titanium dioxide. Here we demonstrate, for the first time, the ability to switch the hydrophilic state of zirconium dioxide surfaces by controlling the spectral composition of the actinic light in the UV spectral range.

## Results

**Figure 1:**
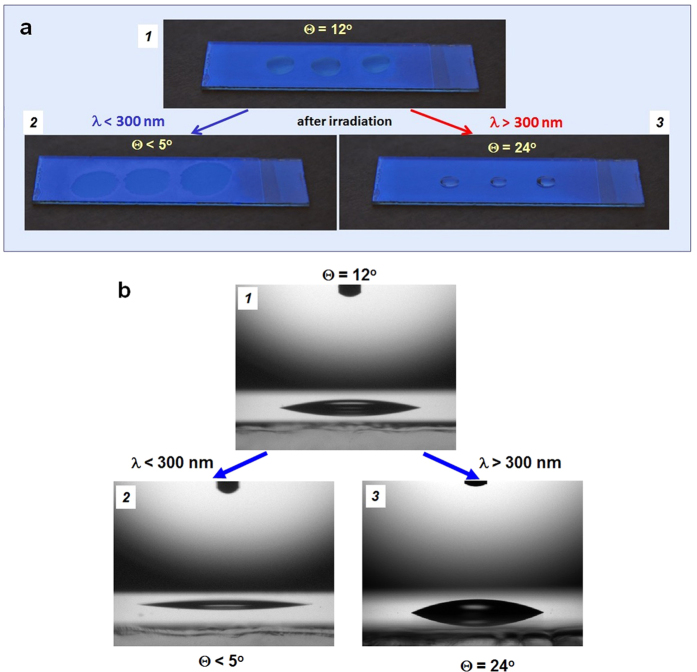
Light-induced hydrophilic behavior of ZrO_2_ films. (**a**) Photo images of water droplets on the ZrO_2_ surface. (**b**) Optical images of water droplets on the ZrO_2_ surface. *1*, An initial hydrophilic state before irradiation. *2*, A superhydrophilic surface after UV-irradiation, λ < 300 nm. *3*, A hydrophobic surface after irradiation by light with λ > 300 nm. Θ is the water contact angle.

The initial state of zirconium dioxide polycrystalline films was characterized by a water contact angle of 12° ± 1° (Fig. 1a,b – image *1*). After irradiation by UV-light with wavelengths shorter than 300 nm the surface became superhydrophilic characterized by a contact angle less than 5° ± 1° (Fig. 1a,b – image *2*). At the same time, irradiation of the initial surface state by light with wavelengths longer than 300 nm results in a significant increase of the water droplet contact angle reaching the value of 24° ± 1° (Fig. 1a,b – image *3*). These variations of the surface hydrophilicity correlate with alteration of the relative signals of O1s in XPS spectra (for details see “Supplementary Information”). In both cases the final surface states after irradiation are reproducible and preserve their hydrophilic state for several hours.

**Figure 2:**
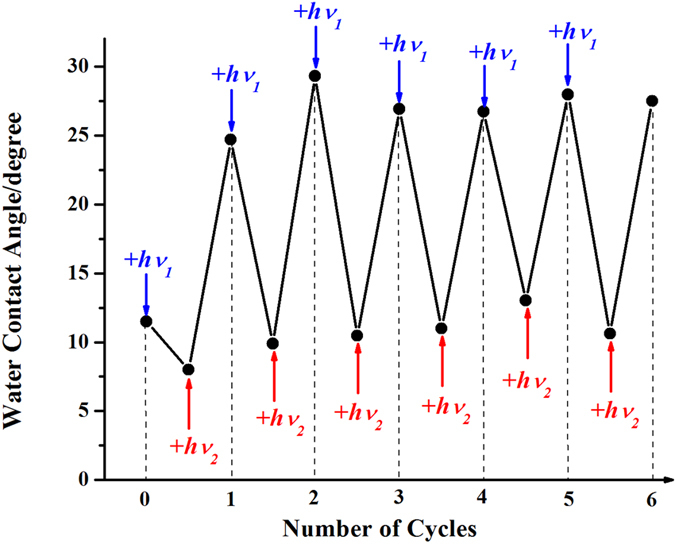
Reversible switching between superhydrophilic and less hydrophilic states of ZrO_2_ by alteration of the spectral composition of actinic light: *hv*_*1*_ corresponds to light with λ < 300 nm, *hv*_*2*_ – to light with λ > 300 nm.

We found that the effect of spectral composition of actinic light on the surface hydrophilicity of zirconia can be reversibly switched from a highly hydrophilic state to a more hydrophobic state. Figure 2 demonstrates the repeatable change of the water contact angle on a ZrO_2_ surface as a result of the alteration of the actinic light spectral composition. In Fig. 2, for simplicity, we present only six cycles of photoinduced switching of the surface hydrophilicity, each cycle consisted of the irradiation by light with λ < 300 nm for 15 minutes and the irradiation by light with λ > 300 nm for 15 minutes. In our experiments, the number of performed cycles reached up to 20 with good reproducibility. Thus, the irradiation in different spectral regions turned the surface of zirconium dioxide film to a superhydrophilic state under irradiation by light with λ < 300 nm, and to a more hydrophobic under irradiation by light with λ > 300 nm, and both states can be repeatedly switched from one to another by alteration of the spectral range of ZrO_2_ photoexcitation.

**Figure 3:**
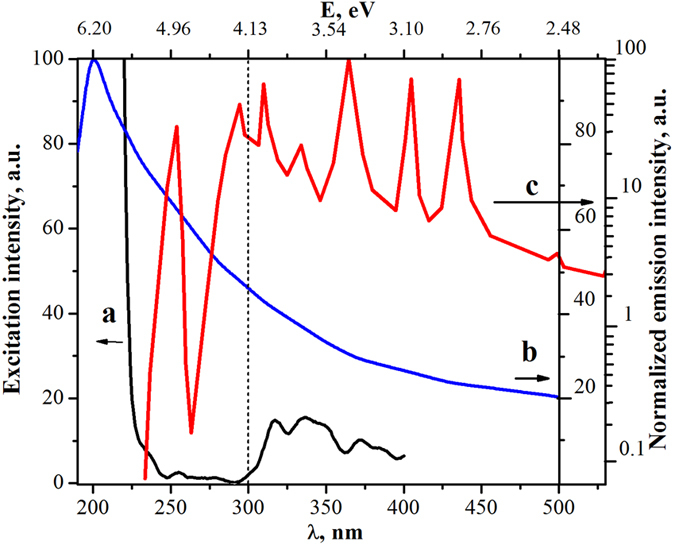
(**a**) Excitation spectrum of ZrO_2_ photoluminescence with emission at 430 nm (2.89 eV). (**b**) Emission spectrum of the 30‒W deuterium lamp DDS-30 (LOMO). (**c**) Emission spectrum of the high-pressure 120-W mercury vapour lamp (LOMO). Emission spectra of the lamps were normalized to an irradiance of 0.1 mWm^−2^ nm^−1^ and 2.4 mWm^−2^ nm^−1^ for deuterium and mercury lamps, respectively. The dashed line shows the position of the cut-off at 300 nm.

It is important to note, that photoexcitation with wavelengths shorter than 300 nm corresponds mostly to the fundamental absorption of zirconia (E_g_ ~ 5.0 eV, *λ* < 250 nm) while irradiation with light λ > 300 nm conforms to extrinsic absorption by defects such as Zr^3+^ and F^+^ -center electronic states in ZrO_2_. This assumption is confirmed by measurements of the photoluminescence excitation spectrum (*λ*_*em*_ *=* 430 nm) presented in Fig. 3 and is in good accordance with earlier spectroscopic and luminescence results^11^.

## Discussion

Luminescence is initiated by intrinsic excitation at energies above 5.0 eV, and by the extrinsic excitation complex band formed by several absorption single bands within the spectral region from ~5 to ~3 eV^11^. Luminescence centers are suggested to be oxygen anion vacancy related defects^11^,^12^,^13^, (V_a_), which behave as deep electron traps:


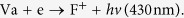


Accordingly, the amphiphilic behavior of zirconia films under light of different spectral composition can be explained by the formation of different active surface states under photoexcitation in the intrinsic and extrinsic absorption regions. In the case of band-to-band excitation, illumination results in the superhydrophilic state. Irradiation in the extrinsic ZrO_2_ region produces a more hydrophobic surface.

**Figure 4:**
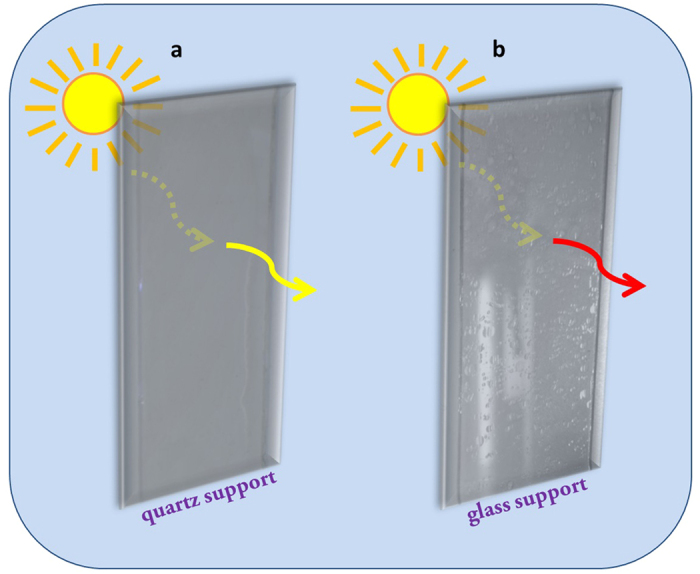
Illustration of photoinduced hydrophilic behavior of two-sided ZrO_2_ coatings on different substrate materials: (**a**) quartz support, (**b**) glass support. Yellow arrow means sunlight. Red arrow indicates the visible part of sunlight. Both sides, towards and away from the sun, of the ZrO_2_ coating on quartz support are superhydrophilic. The back side of the ZrO_2_ coating on glass support is not superhydrophilic in contrast to its front side.

It is important to also note that for two-sided ZrO_2_ coatings, the substrate material plays a significant role in the photoinduced alteration of the surface hydrophilicity for each side. For example, let us consider this effect for ZrO_2_ nano-coatings on quartz and glass supports. The transmittance spectra of both these supports are presented in the “Supplementary Information”. Both sides of two-sided ZrO_2_ films on quartz supports demonstrate an identical photoinduced hydrophilic behavior (Fig. 4a) whatever the spectral composition of actinic light is. UV-light is transmitted through the fused quartz support without absorption, and the nano-coating on both sides becomes hydrophilic. At the same time, the glass support cuts light with λ < 300 nm, and therefore, only one side is irradiated in the intrinsic spectral region while the other side is excited in the extrinsic absorption spectral region only. This results in different hydrophilic behavior of the front and back sides of two-sided ZrO_2_ films on glass support (Fig. 4b): one side transforms to the superhydrophilic state, another to the more hydrophobic state.

The observed light-controllable reversible and reproducible switching of hydrophilicity opens new possible applications for the ZrO_2_ based materials. One of them is the self-cleaning and antifogging effect under ultraviolet irradiation or the raindrop effect under visible light illumination. Another potential application can be oil-water separation. Irradiation by light with wavelengths shorter than 300 nm increases an oil-repellent property of zirconia or zirconia-containing coatings, membranes, and meshes, thus increasing the effectivity of separation or filtration processes of water from organics. There is a commercial ceramic membrane known as a Ceramesh (a composite of a metal mesh and a zirconia ceramic) that is already used for the separation of organics from raw water and water filtration^14^. Our findings could considerably improve the performance of such materials.

## Methods

### ZrO_2_ film preparation

ZrO_2_ nanocoatings were formed by a sol-gel dip coating method (KSV Nima dip coater) onto SiO_2_-coated glass and quartz supports with subsequent annealing at 500 °C in ambient air to remove all organic residuals after synthesis for at least 10 hours. The substrates were pre-cleaned by detergent, sonicated in 2-propanol by using a Branson 5510 ultrasonic cleaner for 25 minutes each and then washed with deionized water and dried at 100 °C. For the SiO_2_ sol, tetraethyl orthosilicate ( ≥99.0% (GC), Aldrich), ethanol (puriss. p.a., absolute, ≥99.8% (GC), Vekton) and nitric acid (aquafortis, ≥99.0%, Vekton) were used as starting material, solvent and stabilizer, respectively. For the ZrO_2_ sol, zirconium (IV) butoxide solution (80 wt% in *1*-butanol, Aldrich), 2-propanol ( ≥99.0%, Vekton) and acetic acid ( ≥98.8%, “pure”, Vekton) were used as starting material, solvent and stabilizer, respectively. The withdrawal velocity from solution was 10 mm.min^−1^ and 20 mm.min^−1^ for SiO_2_ and ZrO_2_ films, respectively.

The thus synthesized ZrO_2_ films were formed by nanoparticles with crystallite size of about 10–15 nm. The film thickness estimated by electronic microscopy was equal to 80–90 nm. Film surface smoothness was determined by AFM to be ± 2nm. Raman spectral analysis detected the presence of two ZrO_2_ phases in the ZrO_2_ coating – tetragonal and monoclinic. XRD analysis is in accordance with the Raman spectroscopic data. The contents of tetragonal and monoclinic phases are 72 ± 3 wt.% and 28 ± 3 wt.%, respectively. Detailed data on the ZrO_2_ coating characterization are presented in the “Supplementary Information”.

### The water contact angle measurement and the study of photoinduced hydrophilic behavior of ZrO_2_ surface

The water contact angle (Θ) values were measured using a Theta Lite optical tensiometer (Biolin Scientific, Finland). Ultrapure water has a pH value of 5.5.

To obtain reproducible initial states of the ZrO_2_ surfaces, we used a special procedure developed in our laboratory and described elsewhere^15^,^16^,^17^ (see also “Supplementary Information”) to obtain a hydrated surface which is characterized by a water contact angle value of 12.0° ± 1°. This state could stay unchanged during several hours at ambient conditions and was used as the initial state for the subsequent set of photo-experiments.

For irradiation in the intrinsic and extrinsic ZrO_2_ regions, a 30‒W deuterium lamp DDS-30 (LOMO) and a high-pressure 120-W mercury lamp (LOMO) were used, respectively. The cut-off filter at 300 nm (LOMO) was used to extract the extrinsic ZrO_2_ region from the light of the Hg lamp.

## Additional Information

**How to cite this article**: Rudakova, A. V. *et al*. Light-Controlled ZrO_2_ Surface Hydrophilicity. *Sci. Rep.* **6**, 34285; doi: 10.1038/srep34285 (2016).

## Supplementary Material

Supplementary Information
