# Supplementary material for: Light-Controlled ZrO2 Surface Hydrophilicity
Source: Sci Rep. 2016 Oct 5;6:34285. doi: 10.1038/srep34285 (PMC5050454; doi:10.1038/srep34285)
Supplement: Supplementary Information [file srep34285-s1.doc]

**Supplementary Information**

Light-Controlled ZrO_2_ Surface Hydrophilicity

Aida V. Rudakova*, Maria V. Maevskaya, Alexei V. Emeline, Detlef W. Bahnemann*

Laboratory “Photoactive Nanocomposite Materials”, Saint-Petersburg State University, Ulyanovskaya str. 1, Peterhof, Saint-Petersburg, 198504 Russia

* corresponding: [aida.rudakova@spbu.ru](mailto:aida.rudakova@spbu.ru), bahnemann@iftc.uni-hannover.de

**ZrO_2_ film pre-treatment**

To get reproducible initial states of the ZrO_2_ surfaces, we used a special procedure developed in our laboratory and described elsewhere^1-3^. Briefly, the studied nano-coatings were treated at 500^o^C for 1 hour and cooled down to ambient temperature in a controlled atmosphere. After this treatment the surface was characterized by a water contact angle value of 3.0^o^±1^o^ (superhydrophilic surface state). At the next step samples were wetted in ultrapure water at pH 5.5 for 30 minutes followed by drying with air flow at 80^o^C to obtain a hydrated surface which is characterized by a water contact angle value of 12.0^o^±1^o^. This state could stay unchanged for several hours under stationary environmental conditions (temperature: +19^o^C, relative humidity: *about* 60 %) and was used as an initial state for the subsequent set of photo-experiments.

**ZrO_2_ film characterization**

## The crystallite size of synthesized ZrO_2_ films and film thickness was estimated by scanning electron microscopy using a Zeiss Supra 40VP microscope. Film surface smoothness was determined by atomic force microscopy (AFM). Synthesized ZrO_2_ films were formed by nanoparticles with crystallite size of about 10-15 nm (Fig. A, B). Film thickness is equal to 80-90 nm (Fig. A). Film surface smoothness is equal ± 2 nm (Fig. B).

**
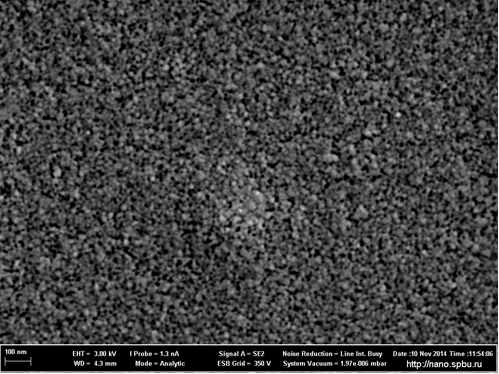

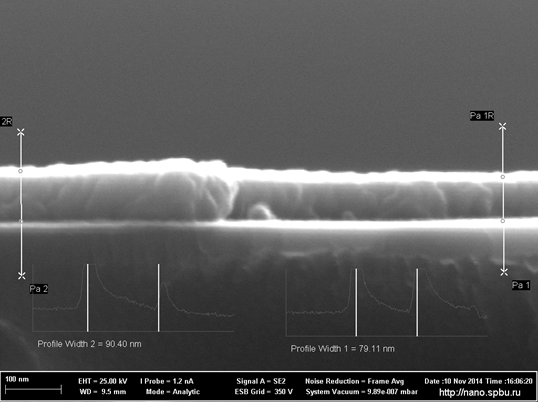
**

Figure A. SEM images of the surface and the cross-section of the ZrO_2_ film on SiO_2_-coated glass support.

**
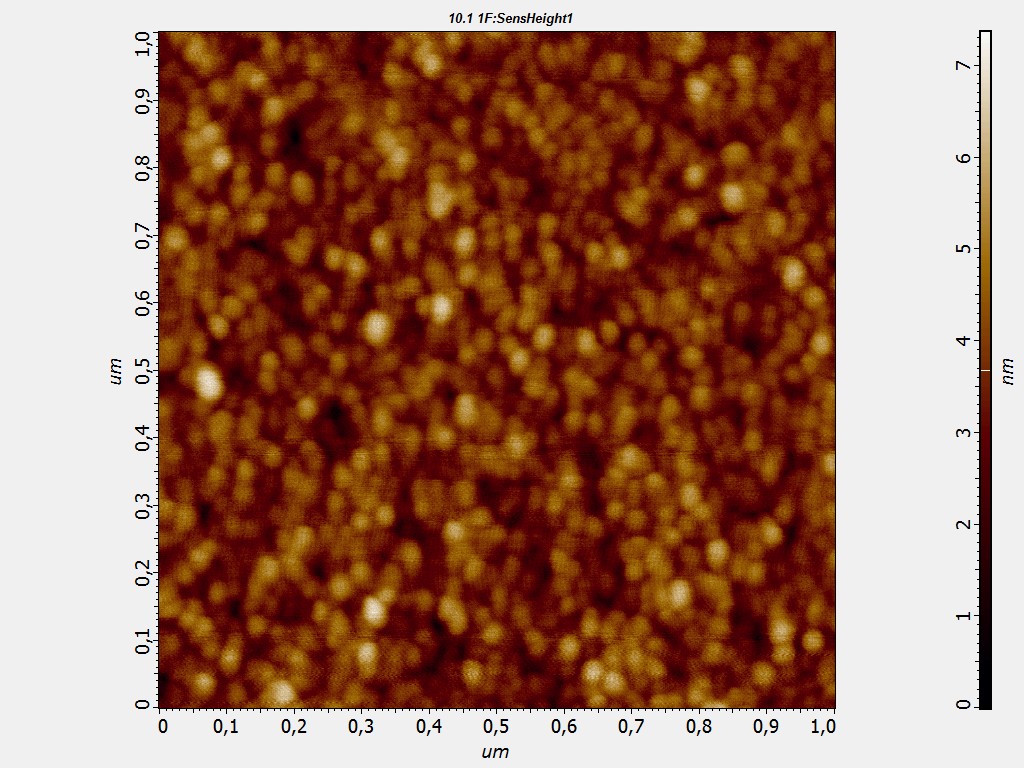

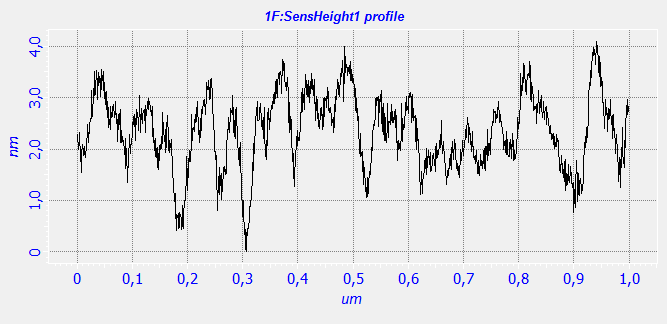
**

Figure B. AFM images of the ZrO_2_ surface profile.

The phase composition of ZrO_2_ nano-coatings thermo-treated at 500^o^C in air was identified using Raman spectroscopy and XRD analysis. The Raman spectrum was registered in the 80-700 cm^-1^ spectral range at ambient temperature using a SENTERRA Raman micro-spectrometer (Bruker, USA) with spectral resolution of 1 cm^-1^ (the excitation laser wavelength was 785 nm, the laser beam power was 100 mW). A Bruker «D8 DISCOVER» high-resolution diffractometer with CuKa radiation in the angle range of 20°≤2θ≤80° with a scanning speed 5.0°/min was used. Structural data was taken from ICSD database.

The Raman spectrum of the ZrO_2_ film with thickness of 90 nm is shown in Figure C. It is seen that two ZrO_2_ phases, tetragonal and monoclinic, are present in the phase composition of the ZrO_2_ coating. The vibrational modes of tetragonal ZrO_2_ are observed at 150 cm^-1^, 269 cm^-1^, 320 cm^-1^, 457 cm^-1^, 476 cm^-1^, and 640 cm^-1^. The bands at 103 cm^-1^, 180 cm^-1^, 190 cm^-1^, 389 cm^-1^, 476 cm^-1^, and 559 cm^-1^ belong to the monoclinic ZrO_2_ phase. Our assignment of characteristic Raman active modes of both ZrO_2_ phases is in accordance with previous data on the characterization of a different ZrO_2_ sample by Raman spectroscopy^4-6^. It is obvious from peak intensities that the tetragonal phase predominates over the monoclinic one.

**
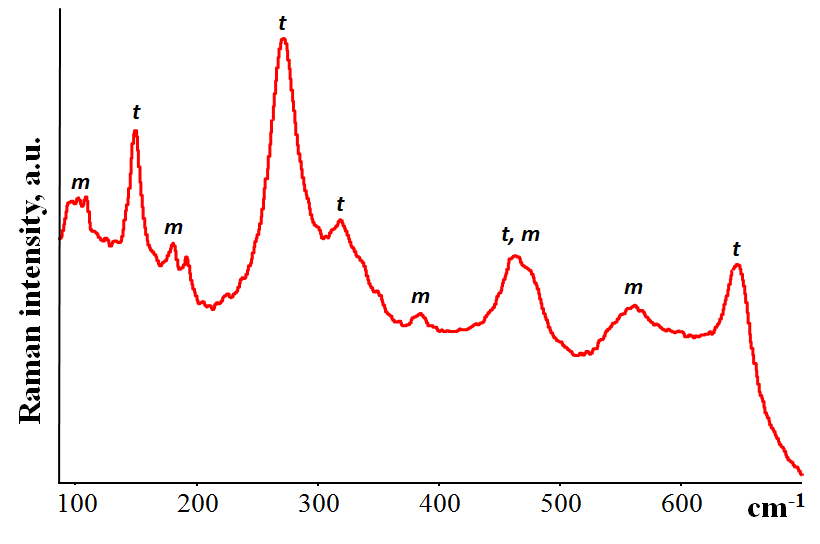
**

Figure C. Raman spectra of ZrO_2_ film on glass support. Peaks marked by *t* belong to the tetragonal ZrO_2_ phase; peaks marked by *m* correspond to the monoclinic ZrO_2_ phase.

The XRD analysis is in agreement with our Raman data. The quantitative analysis was carried out by the Rietveld method. X-ray diffraction patterns of the ZrO_2_ film (Fig. D) and data on the determination of phase composition are presented below. The results of Rietveld analysis show that the contents of tetragonal and monoclinic phases are 72±3 wt.% and 28±3 wt.%, respectively.


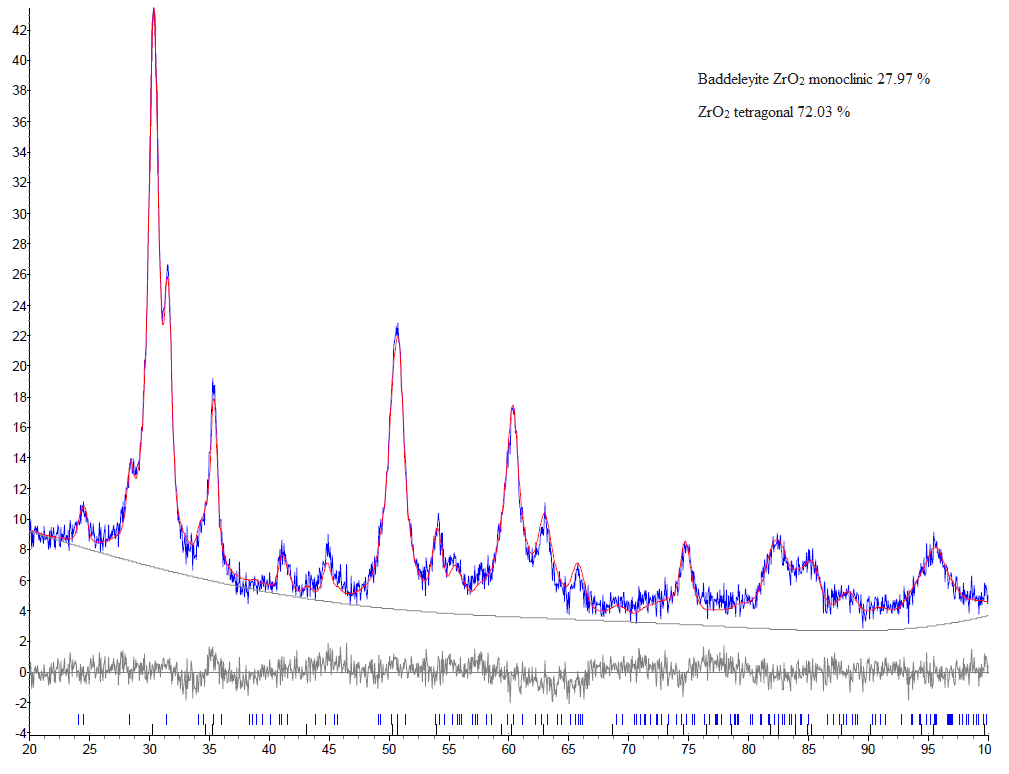


Figure D. X-ray diffraction patterns of the ZrO_2_ film on SiO_2_-coated glass support coating annealed at 500^o^C.

**Analysis of phase composition of the ZrO_2_ film on SiO_2_-coated glass support coating annealed at 500^o^C:**

Quantitative Analysis - Rietveld

Phase 1 : "Baddeleyite ZrO2 monoclinic" 28(3) %

Phase 2 : "ZrO2 tetragonal" 72(3) %

Background

Chebychev polynomial, Coefficient 0 28.2(5)

1 -30.6(7)

2 18.0(5)

3 -5.6(4)

4 2.9(4)

5 0.7(5)

Instrument

Primary radius (mm) 400

Secondary radius (mm) 400

Simple axial model (mm) 2(190)

Additional convolutions

Hat, Constant 0.1435222

Gaussian, 1/Cos(Th) 0.02330084

Lorentzian, 1/Cos(Th) 0.02849391

Circles, Constant 0.1628808

Lorentzian, Constant 0.01697179

Corrections

Specimen displacement -0.2(2)

LP Factor 0

Structure 1

Phase name Baddeleyite ZrO2 monoclinic

R-Bragg 5.510

Spacegroup 14

Scale 0.000117(12)

Cell Mass 492.891

Cell Volume (Е^3) 140.1(3)

Wt% - Rietveld 28(3)

Double-Voigt|Approach

Cry size Lorentzian 0(1400000)

Cry size Gaussian 23(5)

k: 1 LVol-IB (nm) 22(8)

k: 0.89 LVol-FWHM (nm) 21(6)

Strain

Strain L 0(12)

Strain G 1.0(3)

e0 0.003(17)

Crystal Linear Absorption Coeff. (1/cm) 621.0(12)

Crystal Density (g/cm^3) 5.842(11)

Preferred Orientation (Dir 1 : 1 1 -1) 2.6(3)

(Dir 2 : 1 1 1) 0.41(3)

Fraction of Dir 1 0.44(13)

Preferred Orientation Spherical Harmonics

Order 2

y00 1

y20 -0.0(2)

y22m -0.63(12)

y22p -0.05(13)

Lattice parameters

a (Е) 5.134(6)

**XPS data**

The X-ray photoelectron spectroscopy (XPS) measurements were made using an Escalab 250Xi spectrometer (Thermo Fisher Scientific Inc., USA). Figure E shows the XPS spectra of ZrO_2_ nano-films in the O1s region after different treatments: after annealing at 500^o^C in air and wetting in ultrapure water and drying at 80^o^C (taken as an initial state) (a); following irradiation by light with λ<300 nm (b); or following irradiation by light with λ>300 nm (c).

Figure E. XPS spectra of ZrO_2_ nano-films in the O1s region after annealing at 500^o^C in air and wetting in ultrapure water and drying at 80^o^C (a), following irradiation by light with λ<300 nm for 1 hour (b), or following irradiation by light with λ>300 nm for 1 hour (c). Original spectra are in black. Deconvolution was made using Origin 9.0 Software: re-convoluted spectra are colored in cyan, components around 529.8 eV are in red, components around 531.0 eV are in green, and components around 532.3 eV are in blue.

The O1s broad peaks can be deconvoluted into three components with maxima positions at around 529.8 eV, 531.0 eV, and 532.3 eV. The relative contents of these peaks, r_i_, that represents the ratio A_i_/ΣA_i_ (where A_i_ is the area of each peak) are shown in Table A. These three XPS peaks can be attributed to different oxygen species in the ZrO_2_^7-11^. The position of the oxygen O1s peak located at 529.8 eV is due to the presence of reticular oxygen of the zirconia framework. Components around 531.0 eV and 532.3 eV are attributed to Zr-O species and OH groups, respectively. The initial state is a surface state after annealing the films at 500^o^C in the air, wetting in ultrapure water and drying at 80^o^C. The water contact angle values for all these cases are presented for correlation reference.

Table A. XPS peaks for the O1s region and water contact angles for different states of the ZrO_2_ coating surfaces (r_i_ = A_i_/ΣA_i_ ; here A_i_ is the area of each peak).

|  | initial state | after irradiation,  λ<300 nm | after irradiation,  λ>300 nm |
| --- | --- | --- | --- |
| water contact angle (Θ) | 12^0^ | 5^0^ | 24^0^ |
| r_i_(O1s at ~529.8 eV) | 0.79 | 0.74 | 0.78 |
| r_i_(O1s at ~531.0 eV) | 0.20 | 0.13 | 0.20 |
| r_i_(O1s at ~532.3 eV) | 0.01 | 0.13 | 0.02 |

Data presented in Table A shows that after irradiation in the intrinsic ZrO_2_ region (wavelengths shorter than 300 nm) the relative contents of O1s peaks at 529.8 eV and 531.0 eV has decreased, while the r_i_ of O1s peak at 532.3 eV corresponding to the oxygen signal in OH groups has significantly increased. At the same time, the water contact angle fell down to a value of 5^0^, and the ZrO_2_ surface became superhydrophilic. The irradiation of ZrO_2_ nano-coatings in the extrinsic region (wavelengths longer than 300 nm) did not lead to appreciable alterations in O1s peak contents. This influence brought the surface back to the less hydrophilic (more hydrophobic) state with a water contact angle of 24^0^.

**Contact angle measurements for CH_2_I_2_**

CH_2_I_2_ was used as a standard reference for the dispersive component of the surface energy. The results of contact angle measurements for water and methylene iodide are presented in Table B.

Table B. Contact angle values (CA) for water and methylene iodide for ZrO_2_ nano-film on SiO_2_-coated glass support.

|  | CA(H_2_O),^0^ | CA(CH_2_I_2_),^0^ |
| --- | --- | --- |
| initial state | 12±2 | 20±2 |
| after irradiation for 20 min, λ<300 nm | 3±1 | 36±2 |

**Contact angle measurements for bare substrate (SiO_2_-coated glass)**

Table C. Water contact angle (WCA) values for SiO_2_-coated glass support after different treatment.

|  | WCA,^0^ |
| --- | --- |
| after annealing at 500^o^C | 5±1 |
| after wetting in ultrapure water and drying at 80^o^C | 32±2 |
| after irradiation for 20 min, λ<300 nm | 35±2 |

**Transmittance spectra of substrates**

Figure F. Transmittance spectra of substrates: 1 – quartz, 2 – glass.

**References**

1. Rudakova, A. V. *et al.* Photoinduced hydrophilic conversion of hydrated ZnO surfaces. *J. Coll. Interface Sci.* **466**, 452–460 (2016).
2. Emeline, A. V., Rudakova, A. V., Sakai, M., Murakami, T. & Fujishima, A. Factors affecting UV-induced superhydrophilic conversion of a TiO_2_ surface. *J. Phys. Chem. C* **117**, 12086–12092 (2013).
3. [Rudakova](http://pubs.acs.org/author/Rudakova%2C+Aida+V), A. V. *et al.* Dependences of ZnO photoinduced hydrophilic conversion on light intensity and wavelengths. *J. Phys. Chem. C* **119**, 9824–9828 (2015).
4. Jayakumara, S. *et al.* Characterization of nano-crystalline ZrO_2_ synthesized via reactive plasma processing. *Mater. Sci. Eng.: B* **176**, 894–899 (2011).
5. Shi, L., Tin, K.-C. & Wong, N.-B. Thermal stability of zirconia membranes. *J. Mater. Sci.* **34**, 3367–3374 (1999).
6. Siu, G. G., Stokes, M. J. & Liu, Y. Variation of fundamental and higher-order Raman spectra of ZrO_2_ nanograins with annealing temperature. *Phys. Rev. B* **59**, 3173–3179 (1999).
7. Liu, J. *et al.* Low on-resistance diamond field effect transistor with high-κ ZrO_2_ as dielectric. *Sci. Rep.* **4**, 6395 (2014).
8. Han, Y., Yan, Y. & Lu, C. Ultraviolet-enhanced bioactivity of ZrO_2_ films prepared by micro-arc oxidation. *Thin Solid Films* **517**, 1577–1581 (2009).
9. Cubillos, G. I., Olaya, J. J., Bethencourt, M., Cifredo, G. & Blanco, G. Structural changes in ZrO_x_N_y_/ZrO_2_ coatings deposited through spray pyrolysis-nitriding. *Mex. J. Phys.* **60**, 233–242 (2014).
10. Wiame, H., Centeno, M.-A., Picard, S., Bastians, P. & Grange, P. Thermal oxidation under oxygen of zirconium nitride studied by XPS, DRIFTS, TG-MS. *J. Eur. Ceram. Soc.* **18**, 1293-1299 (1998).
11. Basahel, S. N., Ali, T. T., Mokhtar, M. & Narasimharao, K. Influence of crystal structure of nanosized ZrO_2_ on photocatalytic degradation of methyl orange. *Nanosc. Res. Lett.* **10**, 73 (2015).
